# *Mycobacterium avium *subspecies *paratuberculosis *is not associated with Type-2 Diabetes Mellitus

**DOI:** 10.1186/1476-0711-7-9

**Published:** 2008-04-22

**Authors:** Valentina Rosu, Niyaz Ahmed, Daniela Paccagnini, Adolfo Pacifico, Stefania Zanetti, Leonardo A Sechi

**Affiliations:** 1 Dipartimento di Scienze Biomediche, Sezione di Microbiologia clinica e sperimentale, viale San Pietro 43 b 07100 Sassari, Italy; 2 Pathogen Evolution Laboratory, Centre for DNA Fingerprinting and Diagnostics, Hyderabad, India; 3 Servizio di Diabetologia, Clinica Medica Universitaria di Sassari. 07100 Sassari, Italy

## Abstract

**Background:**

The role of pathogenic mycobacteria in diabetes has been a focus of speculation since a decade without any meaningful insights into the mechanism of diabetes causation *vis a vis *mycobacterial factors. Two of our studies based on PCR identification of mycobacterial DNA and detection of antibodies specific to the recombinant antigens and whole cell lysates of the *Mycobacterium avium *subsp. *paratuberculosis *(MAP) shown a clear association of MAP with the presence of type 1 diabetes mellitus (T1DM).

**Methods:**

In this study, we sought to investigate if or not type 2 diabetes (T2DM) patients harbour humoral responses to MAP. Using three different MAP antigen preparations, humoral antibody profiles were estimated for 57 T2DM patients and 57 healthy controls. Statistical analysis was performed with the Chi-square test with Yates' corrections.

**Results:**

We observed insignificant levels of humoral antibodies against recombinant heparin binding haemagglutinin (HbHA), glycosyl transferase (Gsd) and MAP whole cell lysate in the blood of subjects with T2DM as compared to healthy controls.

**Conclusion:**

We found no obvious association of MAP with the incidence of T2DM in Sardinian patients.

## Background

*Mycobacterium avium *subspecies *paratuberculosis *(MAP) is an important pathogen whose role in autoimmune diseases such as Crohn's disease and diabetes has been debated [1-4].

Type 2 diabetes mellitus (T2DM) has become an epidemic, and virtually no physician is without patients who have the disease [5,6]. Adult-onset diabetes mellitus or type 2 diabetes haunts more than 10% of the population in western countries in the age of 30 years old or more [7] and about 20% of people above 75 years of age [8,9]. Moreover, over the last decade, it has become apparent that type 2 diabetes is extending not only to the young adult population but also found in adolescents and even, occasionally, in children [5]. The incidence is on the rise due to increased longevity and life expectancy and change in lifestyles including dietary habits, diminishing physical activity and rampancy of obesity, an increasing trend in many countries [5,6].

Type 1 diabetes mellitus (T1DM) on the other hand is an insulin deficiency syndrome wherein the role of an infectious trigger such as MAP is becoming increasingly evident [10,11]. In our recent studies based on MAP specific DNA and antibody detection [10,11], we observed MAP to be an important link in T1DM in Sardinian diabetic patients who were free of tuberculosis and Crohn's disease.

Previous work [12] demonstrated low levels of antibodies against the 65 kDa heat shock protein (Hsp65) in established T1DM and T2DM cases. Heat shock proteins play an important role in auto-immune diseases and infection [1,8,9,12]. Human glutamic acid decarboxylase (Gad) the prime antigen in Type 1 diabetes has similar amino epitopes as that of Hsp65. Moreover, it is accepted that low levels of Hsp65 antibodies in patients with established diabetes is probably a manifestation of impaired immunity induced by the diabetic state.

In the present study, we show that T2DM patients from Sardinia, in contrast to those with T1DM, do not harbour significant levels of anti Map antibodies in their blood. This finding negates involvement of MAP in T2DM and thereby reaffirms our hypothesis that T1DM (as against T2DM) possibly results from MAP acting as an infectious trigger.

## Methods

A total of 114 participants comprising of 57 patients with T2DM and 57 healthy controls were tested for the presence of MAP specific antibodies. Sera samples were obtained from these subjects after confirming that they were definitely negative for the presence of tuberculosis (negative to PPD and not BCG vaccinated) and autoimmune and genetic diseases other than diabetes. Clinical diagnosis for T2DM was confirmed in the Diabetology Service of the Sassari University Clinics prior to the enrolment of the test subjects. Blood samples were obtained after written informed consents and after approval of the ethics committee of the University of Sassari. Sera samples were made into aliquots and stored at -20°C for short term storage (<6 months) and -80°C for long term storage (>6 months).

Target diagnostic antigens such as recombinant HbHA. Gsd and the whole cell lysates of the MAP bacteria were available from our previous studies [11,13].

Enzyme linked immunosorbent assay (ELISA) was performed to detect humoral response [in test subjects (T2DM) and controls] against the recombinant MAP antigens and the whole cell lysates. Briefly, 5 μg/ml of crude MAP lysate, HbHA and Gsd antigens in carbonate-bicarbonate buffer (14.2 mM Na_2_CO_3_, 34.9 mM NaHCO_3_, 3.1 mM NaN_3_, pH 9.5) were used individually to coat the wells of flat-bottom 96-well immunoplates (Maxisorp; Nunc, Roskilde, Denmark), overnight at 4°C. Next day, plates were blocked for 1 h with 200 μl of 5% non-fat dried milk (SIGMA) and following two washing steps with phosphate buffered saline (PBS) containing 0.05% Tween 20 (PBS-T), serum samples was added at 1:100 dilution. After 2 h of incubation, three washing steps with PBS-T were performed to remove any unbound antibody. A second incubation period followed, and 100 μl of optimally diluted (1:1000) anti-human immunoglobulin G (IgG)-alkaline phosphatase conjugated antibody (Sigma) was added for 1 h at 37°C. Plates were washed four times in PBS-T before 200 μl of p-nitrophenylphosphate (SIGMA FAST™ pNPP tablets Sigma-Aldrich) substrate for alkaline phosphatase (AP) enzyme was added to each well. Plates were incubated at 37°C in the dark until color developed and were read at 405 nm by using a VERSATunable Max microplate reader (Molecular Devices, USA). As a positive control, a T1DM patient's serum sample (No. 14) [11], which was previously found positive for MAP PCR and in ELISA assays for all the three antigens tested (HbHA, Gsd and MAP lysate) was included in each plate. Since Gsd was fused with the affinity tag Maltose binding protein (MBP), sera were tested with the LacZ-MBP control to evaluate a possible seroreactivity. Statistical analysis was performed by the Chi-square test with Yates' correction, *P *values were calculated using the online Graph Pad scientific calculator,  quickcalcs_ttest1.cfm)

## Results and Discussion

**Figure 1:**
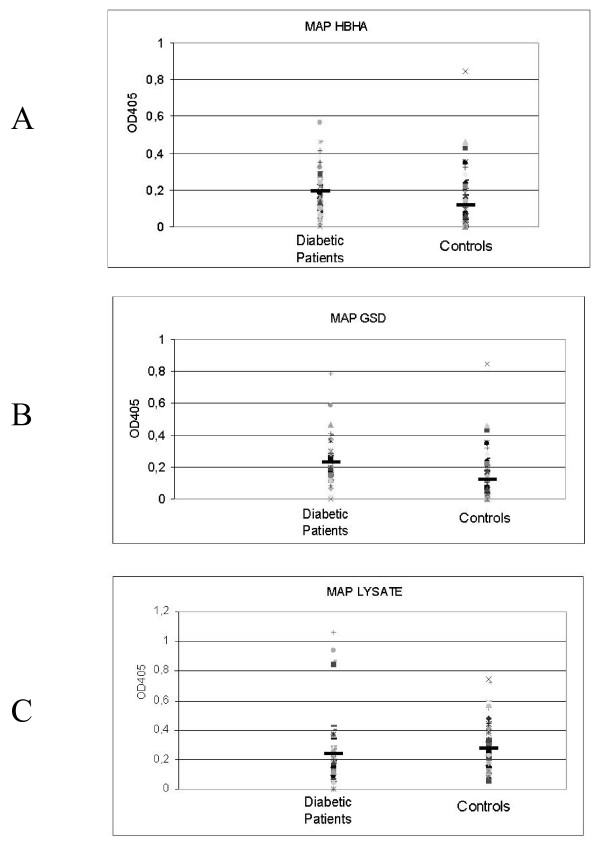
Evaluation of serum samples from diabetic type II patients (left column) and healthy donors (right column) against HbHA recombinant protein (**A**), Gsd recombinant protein (**B**) and MAP lysate (**C**). Data are presented as values of OD405 observed following ELISA, as described in the text. Data from a representative experiment out of three are shown. The median value for each group is indicated by a dark solid horizontal line.

ELISA values were interpreted in the form of optical density (OD) and the values were plotted as shown (Figure 1). An arbitrary cut-off was established as in the previous work [11]. HbHA antigen ELISA titres with a cut-off titre value 0.5 (Chi square equal to 0.000, P value equal to 0.9899); the Gsd protein cut-off value of 0.4 (Chi square equal to 0.154 with 1 degrees of freedom, P value equal to 0.6945); MAP lysate cut-off value of 0.5 (Chi square equal to 0.091 with 1 degrees of freedom, P value equal to 0.7627).

Results of the MAP antigen and whole cell ELISA have been summarized in Figure 1. Overall, the ELISA values were not significant to unravel any relationship between MAP humoral response and the T2DM disease phenotype. This is in contrast to the observations made with T1DM cases where highly significant humoral immune responses to all the three antigens have been documented in the blood of subjects [11]. Also, T1DM patients were shown to harbour circulating loads of bacilli in their peripheral blood as revealed by IS900 based PCR assays [10]. The outcome of the IS900 based PCR assay in our T2DM subjects were also insignificant as seen in case of ELISA. This negates the possibility of a MAP bacteremia in T2DM patients.

MAP is a multi-host pathogen with a broadest host range comprising of many birds, animals and primates [14]. It persists within the human gut in a Ziehl-Neelsen negative "cell wall deficient form" [1,13] which could be the potential source of antigens in the host that may direct auto-immune responses. Deficiency of vitamin D has been implicated as a risk factor for T1DM [8] and T2DM (where vitamin D and calcium insufficiency may negatively influence glycemia), whereas combined supplementation with both the micronutrients may be beneficial in optimizing glucose metabolism [3]. Recently, Vitamin D has been reported to be involved in upregulation of an antimicrobial peptide thus limiting the mycobacterial infections [15]. Such studies possibly help linking the two diseases, diabetes and Crohn's, where MAP could be the common agent putatively behaving as environmental trigger of auto-immunity.

T2DM was linked to mycobacteria as early as in 1943 by Hass and Huntington [16] and afterwards in 1964 [2] and by Schwartz in 1972 [17]. Since then there is a massive body of data that posed speculations on a possible mycobacterial involvement in diabetes [1,8-12,15]. Our results here unequivocally refute such speculations through a robust assay that used three different antigen preparations which were previously shown to be having acceptable sensitivity and specificity in case of T1DM.

## Conclusion

In conclusion, we demonstrate that T2DM patients do not have significant levels of anti MAP antibodies in contrast to their T1DM counterparts. This seemingly hints at the possibility where the involvement of immune dysregulation caused by a persistent pathogen such as MAP (as hypothesized in case of Crohn's disease and T1DM) in T2DM becomes irrelevant as a possible mechanism. At the same time, our observations put at rest the long driven speculation on the involvement of a MAP infection as a trigger in type 2 diabetes.

## Competing interests

The authors declares that they have no competing interests.

## Authors' contributions

VR carried out the carried out protein purification, the immunoassays and performed the statistical analysis. AP participated in recruiting patients and the design of the study. DP was involved in recruiting patients and in processing blood samples. NA participated in study design and drafting the manuscript. SZ participated in the design. LAS designed and coordinated the study and drafted the manuscript. All authors read and approved the final manuscript.
